# Ossification center of the humeral shaft in the human fetus: a CT, digital, and statistical study

**DOI:** 10.1007/s00276-017-1849-4

**Published:** 2017-03-29

**Authors:** Marcin Wiśniewski, Mariusz Baumgart, Magdalena Grzonkowska, Bogdan Małkowski, Arnika Wilińska-Jankowska, Zygmunt Siedlecki, Michał Szpinda

**Affiliations:** 1 Department of Normal Anatomy, The Ludwik Rydygier Collegium Medicum in Bydgoszcz, The Nicolaus Copernicus University in Toruń, Łukasiewicza 1 Street, Bydgoszcz, 85-821 Poland; 2 Department of Positron Emission Tomography and Molecular Imaging, The Ludwik Rydygier Collegium Medicum in Bydgoszcz, The Nicolaus Copernicus University in Toruń, Bydgoszcz, Poland; 3 Clinic of Rheumatology and Systemic Connective Tissue Disorders, J. Biziel University Hospital No. 2, The Ludwik Rydygier Collegium Medicum in Bydgoszcz, The Nicolaus Copernicus University in Toruń, Bydgoszcz, Poland; 4 Department of Neurosurgery, Neurotraumatology and Pediatric Neurosurgery, The Ludwik Rydygier Collegium Medicum in Bydgoszcz, The Nicolaus Copernicus University in Toruń, Bydgoszcz, Poland

**Keywords:** Humeral shaft, Ossification center, Size, Growth dynamics, Human fetus

## Abstract

**Purpose:**

The knowledge of the development of the humeral shaft ossification center may be useful both in determining the fetal stage and maturity and for detecting congenital disorders, as well. This study was performed to quantitatively examine the humeral shaft ossification center with respect to its linear, planar, and volumetric parameters.

**Materials and method:**

Using methods of CT, digital image analysis, and statistics, the size of the humeral shaft ossification center in 48 spontaneously aborted human fetuses aged 17–30 weeks was studied.

**Results:**

With no sex differences, the best-fit growth dynamics for the humeral shaft ossification center was modeled by the following functions: *y* = −78.568 + 34.114 × ln (age) ± 2.160 for its length, *y* = −12.733 + 5.654 × ln(age) ± 0.515 for its proximal transverse diameter, *y* = −4.750 + 2.609 × ln (age) ± 0.294 for its middle transverse diameter, *y* = −10.037 + 4.648 × ln (age) ± 0.560 for its distal transverse diameter, *y* = −146.601 + 11.237 × age ± 19.907 for its projection surface area, and *y* = 121.159 + 0.001 × (age)^4^ ± 102.944 for its volume.

**Conclusions:**

With no sex differences, the ossification center of the humeral shaft grows logarithmically with respect to its length and transverse diameters, linearly with respect to its projection surface area, and fourth-degree polynomially with respect to its volume. The obtained morphometric data of the humeral shaft ossification center are considered normative for respective prenatal weeks and may be of relevance in both the estimation of fetal ages and the ultrasonic diagnostics of congenital defects.

## Introduction

Due to its early and intensive growth, the skeletal system in the fetus may effectively and safely be monitored *in utero* by ultrasound at any period of gestation. However, Victoria et al. [32, 33] reported ultrasonography to be a technique of 40–60% sensitivity in skeletal dysplasias. Thus, low-dose computerized tomography may play a conducive role in cases of suspected fetal skeletal dysplasia, when no specific diagnosis is achieved by ultrasound only [31–33]. Ulla et al. [31] recommended taking up further studies on the clinical use of low-dose CT in fetuses and its risk–benefit analysis. Since the primary ossification centers in the humeral and femoral shafts calcify as early as at week 7 of prenatal life, both may ultrasonically be visualized as the first fetal structures, thus allowing both the assessment of fetal age and detection of potential developmental defects [12]. Although in the assessment of fetal age the priority is given to the femoral length (FL), the humeral length (HL) becomes important for assessing fetal ages in the second and third trimesters of pregnancy in problematic cases [15–17].

Skeletodysplasias display a large and heterogeneous group of genetic defects, in which the defective structure of bones and cartilages is a consequence of their incorrect growth, development, or differentiation. The overall incidence of skeletal dysplasias is 1 case in 5000 live births, which constitutes as many as 5% of children affected by congenital defects [12, 13]. In the diagnostics of skeletodysplasias, a comprehensive identification and evaluation of long bones is indispensable, particularly since in cases of achondroplasia and nanism, dysplasia is limited to one bone only. In the upper limb, dysplasias can affect all bones (micromelia), only the humerus (rhizomelia), the bones of the forearm (mesomelia), or the bones of the hand (acromelia). Diagnosing both rhizomelia and mesomelia requires comparing the size of the appropriate homologous bones in the upper and lower limbs: humerus with femur, radius with tibia, and ulna with fibula [7, 8, 11, 13].

In the present study, we aimed to

- perform morphometric analysis of the humeral shaft ossification center in human fetuses with respect to its linear, planar, and spatial parameters in order to determine their normative specific-age values;
- examine possible differences between sexes for all analyzed parameters; and
- compute growth dynamics for the analyzed parameters, expressed by best-matched mathematical models.

## Materials and methods

The study material comprised 48 human fetuses of both sexes (26 males and 22 females) aged 17–30 weeks, originating from spontaneous abortions or preterm deliveries. All fetuses were preserved by immersion in 10% neutral formalin solution. Fetal ages were previously established on the base of the specimen’s crown–rump length [3]. Each crown–rump length measurement was performed by one researcher but three times under the same conditions at different times, and then averaged. The material was acquired before the year 2000 and remains part of the specimen collection of our Department of Normal Anatomy. The experiment was approved by the Bioethics Committee of our University (KB 275/2011). Table 1 lists the characteristics of the study group, including age, number, and sex of the fetuses.

**Table 1:** Age, number, and sex of the fetuses studied

Gestational age	Crown–rump length (mm)				Number of fetuses	Sex	
Weeks (Hbd-life)	Mean	SD	Min	Max		♂	♀
17	116.00	1.00	115.0	117.0	3	1	2
18	133.33	5.77	130.0	140.0	3	1	2
19	150.60	2.97	146.0	154.0	5	2	3
20	159.00	1.00	158.0	160.0	3	2	1
21	174.75	2.87	171.0	178.0	4	3	1
22	184.00	1.41	183.0	185.0	2	1	1
23	196.33	1.15	195.0	197.0	3	1	2
24	209.33	3.44	205.0	213.0	6	4	2
25	214.33	1.53	213.0	216.0	3	1	2
26	230.33	4.62	225.0	233.0	3	1	2
27	238.40	2.79	235.0	241.0	5	5	0
28	249.50	0.71	249.0	250.0	2	1	1
29	253.00	0.00	253.0	253.0	2	0	2
30	263.25	1.26	262.0	265.0	4	3	1
Total					48	26	22

**Fig. 1:**
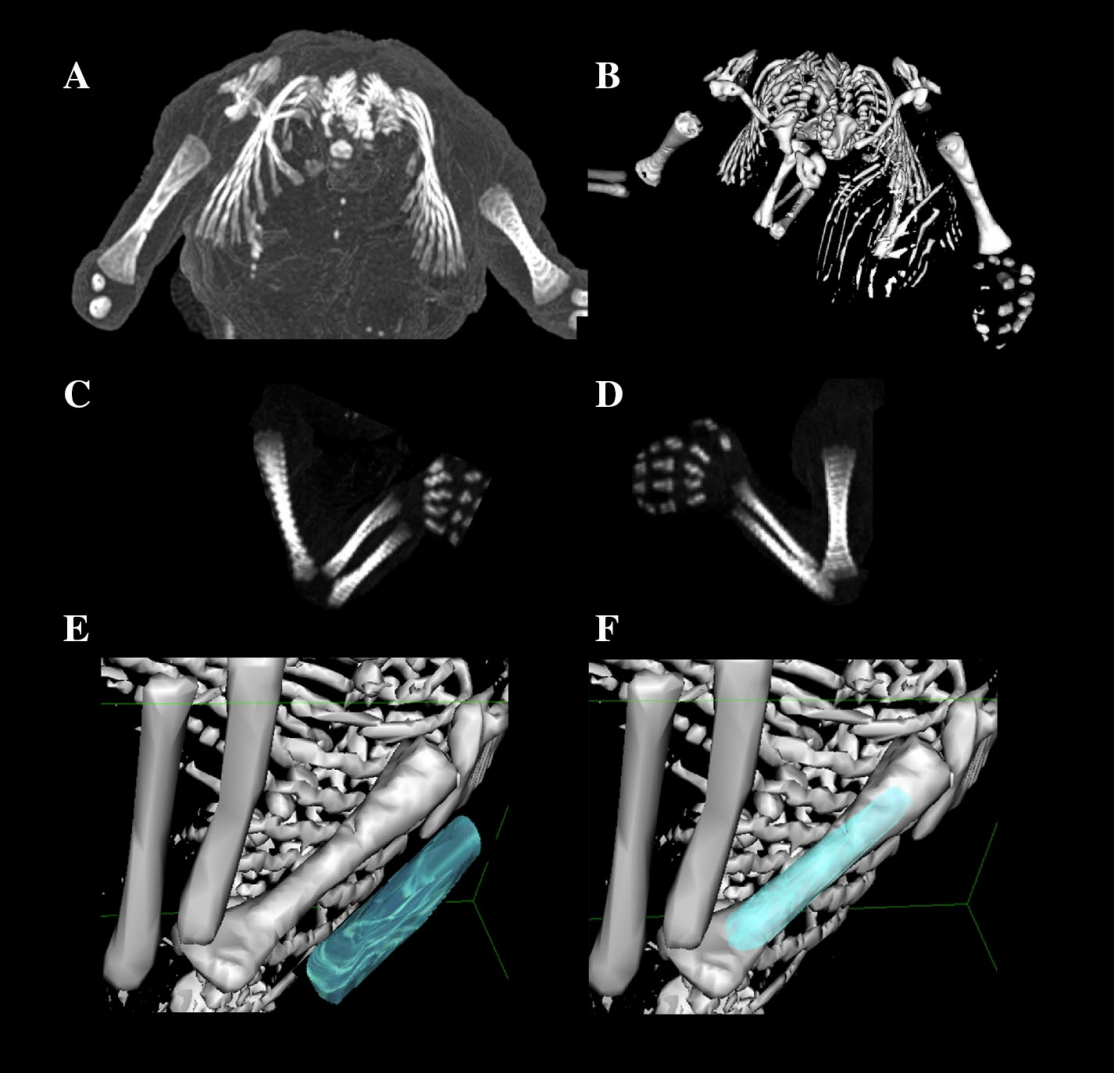
A male human fetus aged 21 weeks in the transverse projection (**a**), its skeletal reconstruction (**b**), its *right* and *left* upper limbs in the lateral projection (**c, d**), its visualization referring to the left humerus (**e**), and humeral shaft ossification center (**f**) using OsiriX 3.9

Using the Siemens Biograph 128 mCT camera, the fetuses were scanned at a step of 0.4 mm, recorded in DICOM formats (Fig. 1), and subsequently subjected to morphometric analysis with the use of the OsiriX 3.9 software. It should be emphasized that OsiriX 3.9 permits precise numerical analysis of any type of linear, planar, and three-dimensional reconstructions of the studied objects.

The gray scale in Hounsfield units of achieved CT pictures ranged from −275 to −134 for a minimum, and from +1165 to +1558 for a maximum. Thus, the window width (WW) alternated from 1.404 to 1.692, and the window level (WL) varied from +463 to +712. The details of the imaging protocol were as follows: mAs—60, kV—80, pitch—0.35, FoV—180, and rot. time—0.5 s, while the details of CT data were as follows: slice thickness—0.4 mm, image increment—0.6 mm, and kernel—B45 f-medium.

Despite the cartilaginous stage, contours of the proximal and distal ends of the humeral shaft ossification center were already clearly visible, thus enabling a precise morphometric analysis of its linear, planar, and volumetric parameters [3].

**Fig. 2:**
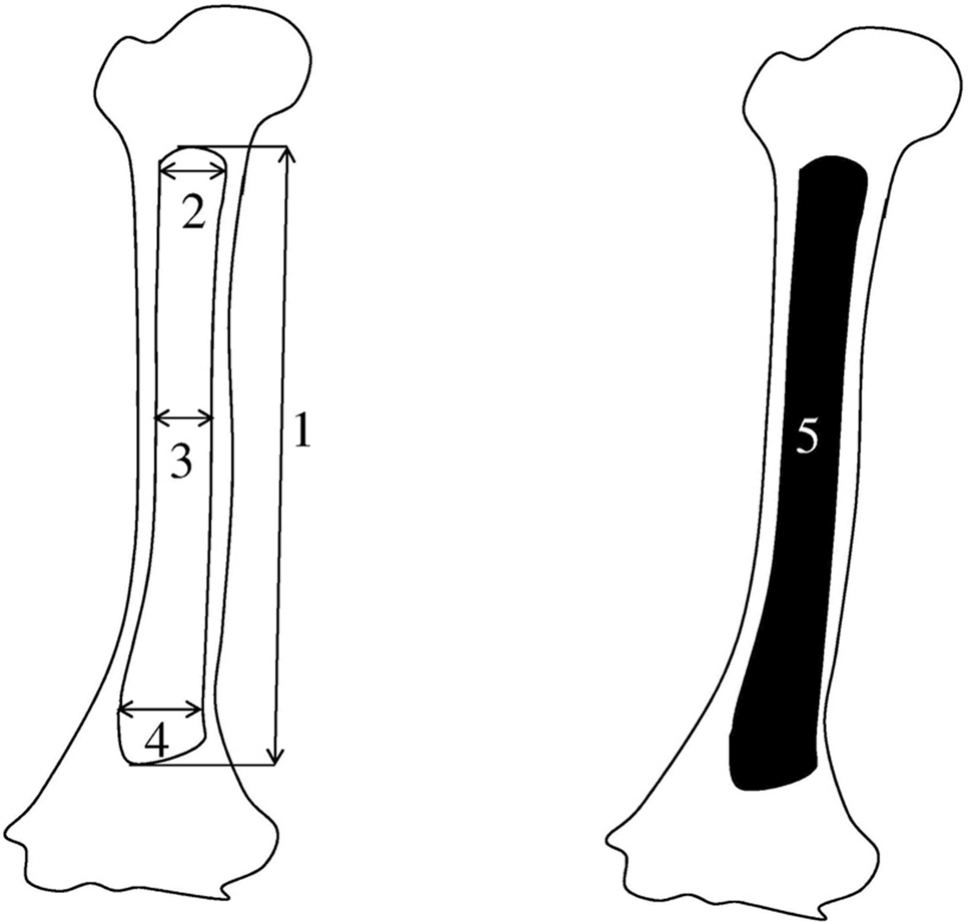
Measurement scheme of the humeral shaft ossification center in the frontal plane. *1* length, *2* proximal transverse diameter, *3* middle transverse diameter, *4* distal transverse diameter, *5* projection surface area

Measurements of the humeral shaft ossification center were conducted in a specific sequence (Fig. 2). In each fetus, the assessment of linear diameters, projection surface area, and volume of the humeral shaft ossification center was carried out. In all, the following six parameters of the humeral shaft ossification center were evaluated:

1. length, based on the determined distance between the proximal and distal borderlines of the ossification center in the frontal plane (Fig. 2);
2. proximal transverse diameter, based on the determined distance between the medial and lateral borderlines of the proximal region of the ossification center in the frontal plane (Fig. 2);
3. middle transverse diameter, based on the determined distance between the medial and lateral borderlines of the central region of the ossification center in the frontal plane (Fig. 2);
4. distal transverse diameter, based on the determined distance between the medial and lateral borderlines of the distal region of the ossification center in the frontal plane (Fig. 2);
5. projection surface area, based on the determined contour of the humeral shaft ossification center in the frontal plane (Fig. 2); and
6. volume, calculated using advanced diagnostic imaging tools for 3D reconstruction, taking into account the position and the absorption of radiation by bone tissue (Fig. 1f).

In an incessant attempt to minimize measurement and observer bias, all measurements were completed by one experienced researcher (MW), specializing in image interpretation. Each measurement was reiterated three times under the same conditions but at different times, and then averaged. The intra-observer variation was assessed by the one-way ANOVA test for paired data. The study results were statistically analyzed. Distribution of variables was checked using the Shapiro–Wilk test, while homogeneity of variance was checked using Fisher’s test. The results have been expressed as arithmetic means with standard deviation (SD), minimal (Min.), and maximal (Max.) values. To compare the means, Student’s t test for independent variables and one-way ANOVA were used. Tukey’s test was used for post hoc analysis. If no similarity of variance occurred, the non-parametric Kruskal–Wallis test was used. The characterization of developmental dynamics of the analyzed parameters was based on linear and curvilinear regression analysis. The match between the estimated curves and measurement results was evaluated based on the coefficient of determination (R^2^).

## Results

No statistically significant differences (*P* > 0.05) in evaluating intra-observer reproducibility of measures of the humeral shaft ossification centers were found. Mean, standard deviations, and minimal and maximal values of all the analyzed parameters of the left and right humeral shaft ossification centers in human fetuses at varying gestational ages are presented in Tables 2 and 3 for length, proximal, middle, and distal transverse diameters and in Table 4 for projection surface area and volume.

**Table 2:** Length and transverse diameters for proximal end, middle part, and distal end of the right humeral shaft ossification center in human fetuses

Gestational age (weeks)	*N*	Length				Transverse diameter (mm)											
						Proximal				Middle				Distal			
		Mean	SD	Min.	Max.	Mean	SD	Min.	Max.	Mean	SD	Min.	Max.	Mean	SD	Min.	Max.
17	3	19.52	0.02	19.50	19.53	3.71	0.02	3.70	3.73	2.85	0.02	2.84	2.87	3.81	0.03	3.79	3.84
18	3	20.30	1.03	19.43	21.43	3.41	0.40	3.16	3.87	2.82	0.06	2.76	2.87	3.47	0.35	3.24	3.87
19	5	20.37	1.54	19.27	23.04	3.37	0.31	3.06	3.70	2.89	0.21	2.65	3.21	3.19	0.91	2.12	4.26
20	3	20.78	0.01	20.77	20.79	3.92	0.01	3.92	3.93	2.93	0.01	2.92	2.94	3.61	0.23	3.45	3.87
21	4	24.97	2.09	22.96	27.81	4.81	0.39	4.37	5.25	3.20	0.13	3.08	3.34	4.35	0.69	3.54	4.94
22	2	26.77	1.29	25.85	27.68	4.14	0.13	4.05	4.23	3.25	0.40	2.96	3.53	4.26	0.68	3.78	4.74
23	3	29.54	1.91	27.63	31.45	5.01	0.75	4.14	5.44	3.28	0.26	3.05	3.56	4.69	0.77	4.03	5.54
24	6	30.31	1.72	28.02	32.44	5.33	0.54	4.53	5.98	3.50	0.23	3.28	3.92	4.61	0.40	4.03	5.28
25	3	29.10	0.20	28.90	29.30	5.10	0.20	4.90	5.30	3.85	0.03	3.83	3.88	4.63	0.02	4.61	4.65
26	3	32.13	2.35	29.51	34.07	6.04	0.49	5.63	6.59	3.55	0.15	3.38	3.65	5.23	0.69	4.59	5.96
27	5	33.10	1.99	30.05	34.94	5.99	0.53	5.30	6.48	3.72	0.27	3.47	4.10	5.53	0.48	5.07	6.06
28	2	36.95	0.02	36.93	36.96	5.80	0.02	5.78	5.81	3.74	0.02	3.72	3.75	5.40	0.14	5.30	5.50
29	2	34.39	1.47	33.35	35.43	6.02	0.11	5.94	6.09	3.90	0.17	3.78	4.02	5.13	0.04	5.10	5.15
30	4	36.40	3.08	32.78	39.05	6.23	0.57	5.64	6.87	3.86	0.22	3.68	4.18	5.69	0.56	5.04	6.42

**Table 3:** Length and transverse diameters for proximal end, middle part, and distal end of the left humeral shaft ossification center in human fetuses

Gestational age (weeks)	*N*	Length				Transverse diameter (mm)											
						Proximal				Middle				Distal			
		Mean	SD	Min.	Max.	Mean	SD	Min.	Max.	Mean	SD	Min.	Max.	Mean	SD	Min.	Max.
17	3	19.53	0.03	19.51	19.56	3.91	0.02	3.90	3.93	2.74	0.01	2.73	2.75	3.43	0.02	3.41	3.44
18	3	21.01	2.24	19.49	23.58	3.46	0.74	2.93	4.30	2.58	0.08	2.48	2.63	3.26	0.12	3.12	3.34
19	5	20.05	1.35	19.18	22.43	3.36	0.36	2.93	3.76	2.59	0.21	2.30	2.80	3.57	0.30	3.14	3.96
20	3	21.33	0.01	21.32	21.34	4.08	0.01	4.07	4.09	2.90	0.01	2.89	2.91	3.06	0.01	3.06	3.07
21	4	22.72	2.97	20.35	26.72	4.63	0.35	4.14	4.88	3.47	0.36	3.07	3.84	4.29	0.84	3.40	5.03
22	2	27.06	1.43	26.05	28.07	4.11	0.11	4.03	4.18	3.34	0.37	3.08	3.60	4.31	0.32	4.08	4.53
23	3	29.75	1.54	28.18	31.25	5.07	0.68	4.29	5.57	3.20	0.13	3.07	3.32	4.45	0.36	4.04	4.68
24	6	30.59	2.31	27.59	33.62	5.30	0.48	4.64	5.80	3.51	0.31	3.06	3.85	4.40	0.28	3.90	4.67
25	3	35.01	0.03	34.98	35.04	5.45	0.03	5.43	5.48	4.13	0.03	4.10	4.15	4.56	0.03	4.53	4.58
26	3	32.87	1.78	31.21	34.75	6.01	0.55	5.63	6.64	4.14	0.36	3.78	4.49	4.97	0.92	4.30	6.02
27	5	33.67	1.81	31.38	35.44	6.42	0.66	5.39	7.01	3.98	0.40	3.45	4.47	5.87	0.73	5.07	6.54
28	2	36.63	0.02	36.61	36.64	6.10	0.01	6.09	6.11	4.16	0.01	4.15	4.17	6.10	0.01	6.09	6.11
29	2	35.60	0.73	35.08	36.11	6.25	0.04	6.22	6.27	4.38	0.64	3.93	4.83	5.29	0.10	5.22	5.36
30	4	37.42	2.07	35.23	39.59	6.27	0.44	5.76	6.70	4.00	0.17	3.84	4.22	5.84	0.61	5.09	6.47

**Table 4:** Projection surface area and volume of the right and left humeral shaft ossification centers

Gestational age (weeks)	*N*	Projection surface area (mm^2^)								Volume (mm^3^)							
		Right				Left				Right				Left			
		Mean	SD	Min.	Max.	Mean	SD	Min.	Max.	Mean	SD	Min.	Max.	Mean	SD	Min.	Max.
17	3	53.13	0.25	52.90	53.40	41.33	0.15	41.20	41.50	224.73	0.42	224.40	225.20	228.47	0.31	228.20	228.80
18	3	56.73	13.86	46.50	72.50	52.53	7.38	46.60	60.80	165.13	62.75	102.50	228.00	172.27	60.88	114.70	236.00
19	5	60.00	5.57	52.50	65.30	56.00	12.55	34.80	66.50	214.18	100.56	109.60	380.90	243.82	129.13	131.10	466.70
20	3	61.10	0.10	61.00	61.20	65.80	0.10	65.70	65.90	325.00	1.00	324.00	326.00	318.17	0.12	318.10	318.30
21	4	92.85	27.08	64.70	125.60	90.25	23.67	67.80	117.30	332.13	130.18	216.90	513.90	336.05	117.82	223.10	496.40
22	2	109.15	23.55	92.50	125.80	109.65	25.10	91.90	127.40	337.65	41.79	308.10	367.20	327.35	26.09	308.90	345.80
23	3	121.03	11.74	109.90	133.30	118.80	17.25	108.20	138.70	374.67	69.06	298.00	432.00	406.00	126.49	306.00	548.20
24	6	127.50	11.03	106.20	137.40	124.57	14.94	95.40	138.60	494.40	138.62	322.80	680.70	500.65	137.40	349.70	683.10
25	3	105.57	0.25	105.30	105.80	104.73	0.40	104.30	105.10	690.43	0.25	690.20	690.70	689.00	2.00	687.00	691.00
26	3	169.60	23.49	142.80	186.60	162.60	8.16	157.30	172.00	633.47	81.21	554.80	717.00	721.60	156.92	566.00	879.80
27	5	162.88	20.44	133.70	184.50	169.74	15.56	155.70	195.70	656.06	112.85	567.80	851.70	718.80	148.32	577.90	902.30
28	2	195.35	0.21	195.20	195.50	189.00	2.83	187.00	191.00	636.00	1.41	635.00	637.00	858.55	0.21	858.40	858.70
29	2	149.20	13.72	139.50	158.90	130.20	2.55	128.40	132.00	747.60	53.03	710.10	785.10	841.20	47.94	807.30	875.10
30	4	177.65	35.41	148.40	225.00	179.53	33.32	152.90	222.00	967.68	5.65	961.00	972.70	966.38	51.49	911.90	1024.40

The statistical analysis revealed neither significant sex nor bilateral differences, which allowed us to compute one growth curve for each analyzed parameter. On both the left and right sides, the growth dynamics of the length and three transverse diameters of the humeral shaft ossification centers followed a natural logarithmic function.

**Fig. 3:**
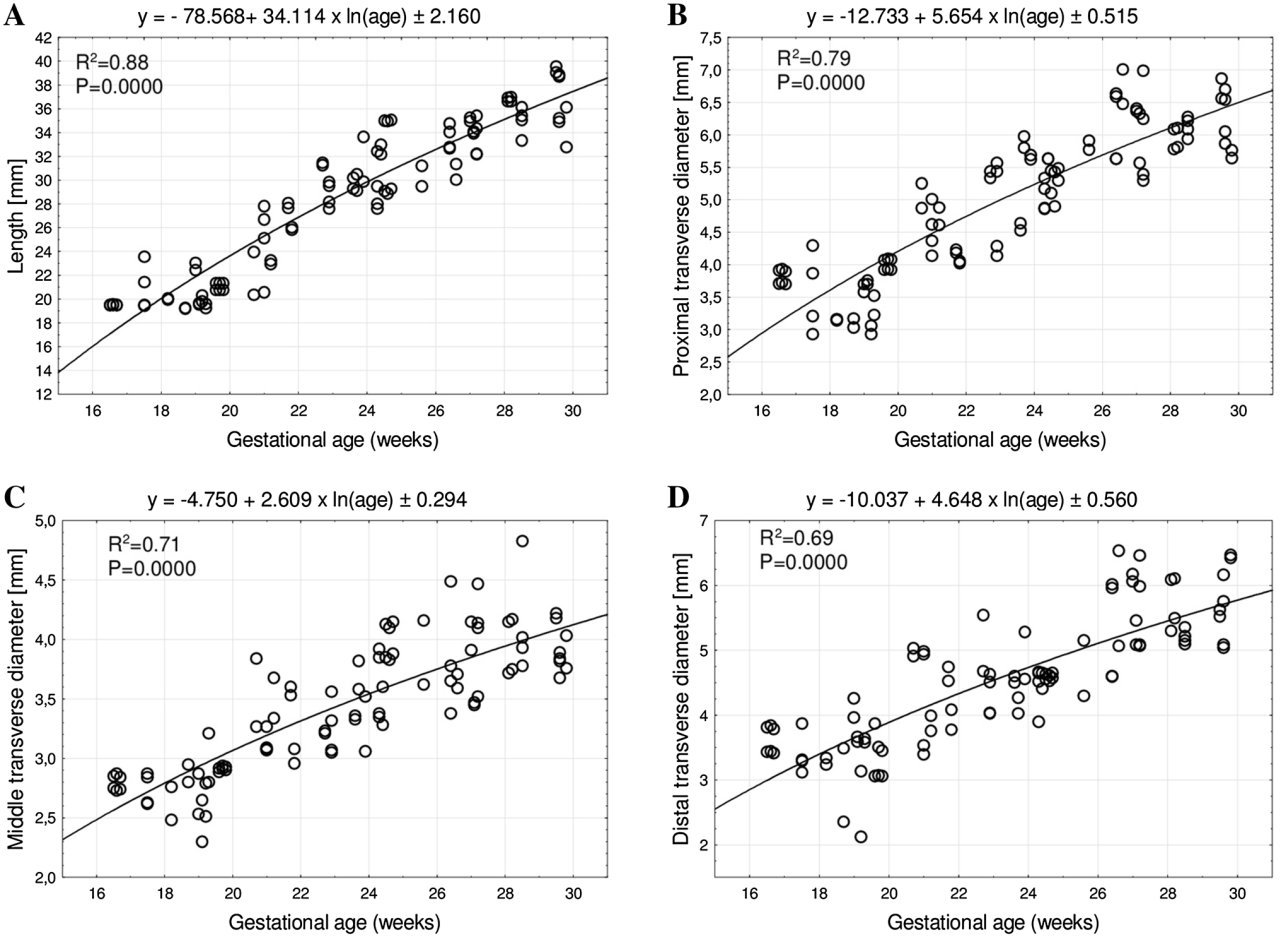
Regression lines for length (**a**), proximal (**b**), middle (**c**), and distal (**d**) transverse diameters of the humeral shaft ossification center

The mean length of the humeral shaft ossification center at fetal ages of 17–30 weeks increased from 19.52 ± 0.02 to 36.40 ± 3.08 mm on the right, and from 19.53 ± 0.03 to 37.42 ± 2.07 mm on the left, following the logarithmic function y = − 78.568 + 34.114 × ln(age) ± 2.160 (*R* ^2^ = 0.88) – (Fig. 3a).

Between weeks 17 and 30, the mean proximal transverse diameter of the humeral shaft ossification center ranged from 3.71 ± 0.02 to 6.23 ± 0.57 mm on the right side, and from 3.91 ± 0.02 to 6.27 ± 0.44 mm on the left side, following the logarithmic function *y* = −12.733 + 5.654 × ln (age) ± 0.515 (*R* ^2^ = 0.79)—(Fig. 3b). The mean middle transverse diameter of the humeral shaft ossification center ranged from 2.85 ± 0.02 mm at 17 weeks to 3.86 ± 0.22 mm at 30 weeks on the right, and from 2.74 ± 0.01 to 4.00 ± 0.17 mm on the left, respectively, following the logarithmic function *y* = −4.750 + 2.609 × ln (age) ± 0.294 (*R* ^2^ = 0.71)—Fig. 3c). In fetuses aged 17–30 weeks, the mean distal transverse diameter of the humeral shaft ossification center ranged from 3.81 ± 0.03 to 5.69 ± 0.56 mm on the right, and from 3.43 ± 0.02 to 5.84 ± 0.61 mm on the left, in accordance with the logarithmic function: *y* = −10.037 + 4.648 × ln (age) ± 0.560 (*R* ^2^ = 0.69)—(Fig. 3d).

**Fig. 4:**
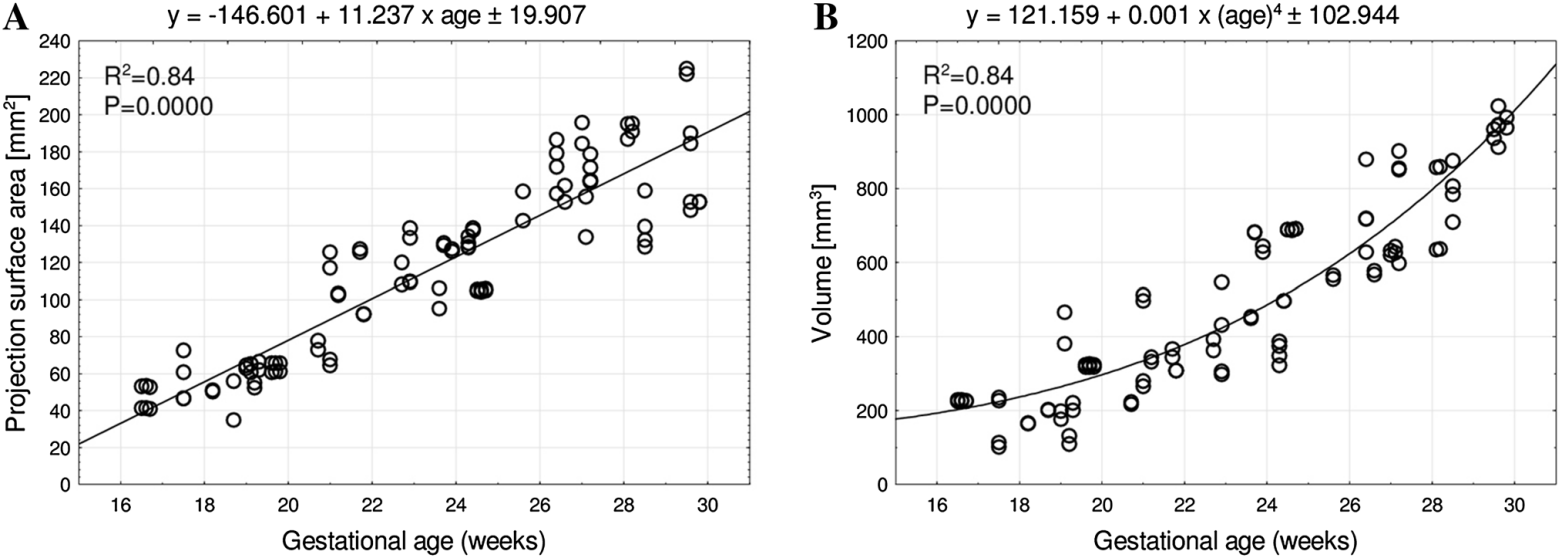
Regression lines for projection surface area (**a**) and volume (**b**) of the humeral shaft ossification center

The mean projection surface area of the humeral shaft ossification center ranged from 53.13 ± 0.25 mm^2^ at 17 weeks to 177.65 ± 35.41 mm^2^ at 30 weeks on the right, and from 41.33 ± 0.15 mm^2^ to 179.53 ± 33.32 mm^2^ on the left side, respectively, following the linear function: *y* = −146.601 + 11.237 × age ± 19.907 (*R* ^2^ = 0.84) (Fig. 4a).

In the fetal age range of 17–30 weeks, the mean volume of the humeral shaft ossification center increased from 224.73 ± 0.42 to 967.68 ± 5.65 mm^3^ on the right side, and from 228.47 ± 0.31 to 966.38 ± 51.49 mm^3^ on the left side, following the fourth-degree polynomial function: y = 121.159 + 0.001 × (age)^4^ ± 102.944 (*R* ^2^ = 0.84)—(Fig. 4b).

## Discussion

In human fetuses, the process of ossification starts earlier in the upper limb than in the lower limb, and consecutively involves the humerus, radius and ulna, metacarpal bones, and phalanges [27]. According to numerous authors [15, 18, 19, 25], the commencement of ossification depends on the fetal body weight, maturity, sex, and ethnic origin. The presence of ossification center in the proximal region of the humerus was confirmed radiologically in 15% fetuses aged 38–39 weeks, in 40% fetuses aged 40–41 weeks, and in 82% fetuses beyond 42 weeks [19]. In turn, a constant presence of the ossification center in question was revealed by Nazario et al. [26] using ultrasound in fetuses at 38 weeks, whereas Donne et al. [16] observed it only in 28% fetuses aged 38 weeks, in 39% fetuses aged 39 weeks, and in 55% fetuses aged 40 weeks. Using ultrasound, Mahony et al. [23] reported the appearance of the ossification center in the proximal region of the humerus as late as at 38 weeks of gestation, while Kumari et al. [20] ultrasonically visualized it as early as at 35–36 weeks of gestation. Of note, in our study involving fetuses at the age of 17–30 weeks, the use of CT permitted an accurate visualization of the humeral shaft ossification center in all the fetuses examined.

In newborns, the humerus is ossified only in its shaft, which constitutes approximately 79% of the entire bone, while its rounded proximal epiphysis and triangular distal epiphysis are still cartilaginous. The proximal end of the humerus develops from the three secondary ossification centers located in its head, greater and lesser tubercles [34], with the fusion of these ossification centers starting at the age of 3 years and progressing in the anteroposterior direction. The humeral head ossification center appeared between the age of 6 and 20 months, while the ossification centers in the greater and lesser tubercles appeared between the age of 1 and 3 years [1]. However, earlier ossification was reported in a radiographic study by Ogden et al. [28] in human cadavers ranging from full-term stillborn to fourteen years old, in which the ossification of the humeral head began between the age of 2 and 3 months, while that of the greater tubercle began at the age of roughly 7 months. This fact was confirmed in an MRI study by Kwong et al. [21], who demonstrated that ossification within the humeral head and greater tubercle occurred at the age of 4 and 10 months, respectively. Broker and Burbach [9] reported the proximal epiphysis of the humerus to ossify in 15–20% neonates younger than 39 weeks and in 40% neonates younger than 41 weeks. The presence of the third ossification center in the lesser tubercle of humerus is rather controversial. Cocchi et al. [14] found the ossification center in the lesser tubercle to appear as late as at the age of approximately 5 years and to subsequently fuse with the ossification centers of the head and greater tubercle at the age of 6–7 years. Furthermore, with the use of MRI in children aged 2 months to 17 years, Kwong et al. [21] observed that there was no separate ossification center in the lesser tubercle. Instead, it was just the humeral head ossification center that also progressively invaded the lesser tubercle. The hypothesis of the two ossification centers in the proximal epiphysis of the humerus was also supported by Paterson [29] and Ogden et al. [28]. Secondary ossification centers appeared more rapidly in girls than in boys since the fusion of the humeral shaft with the proximal end occurred between the age of 12 and 19 years in females and between the age of 15.5 and 20 years in males [34]. Separation of the proximal humeral epiphysis (SPHE) is a common complication observed in neonates after difficult delivery [17].

The distal end of the humerus ossifies from four ossification centers. The first ossification center appears at the age of 6–12 months within the capitulum, the second one appears at the age of 5–7 years in the medial epicondyle, the third one appears at the age of approximately 10 years in the trochlea, and the fourth one appears at the age of 12–14 years in the lateral epicondyle [11, 34]. The fusion of these four ossification centers occurs at the age of 10–12 years, while that of the distal end with the humeral shaft occurs at the age of approximately 15 years. The medial epicondyle first fuses with the humeral shaft and subsequently with the remaining three ossification centers of the distal end between the age of 11 and 16 years in females and between the age of 14 and 19 years in males [34].

This paper is the first report in the medical literature concerning the quantitative analysis of the humeral shaft ossification center in human fetuses, precisely expressed by best-fit mathematical growth models. Of note, the examined ossification center demonstrated neither sexual nor bilateral differences, which clearly corresponded with previous observations presented by Baumgart et al. [3] with relation to the ossification center of the clavicle. Both the length and transverse diameters of the humeral shaft ossification center followed natural logarithmic functions, with fetal ages expressed in weeks. The present study also revealed the projection surface area of the humeral shaft ossification center to commensurately increase with age. In turn, the volume of the humeral shaft ossification center followed the fourth-degree polynomial function. It should be emphasized that the growth dynamics of the humeral shaft ossification center was analogous to that of the clavicle ossification center, since the latter grew logarithmically with respect to its length (*y* = −31.373 + 15.243 × ln (age) ± 1.424) and transverse diameters (*y* = −7.945 + 3.225 × ln (age) ± 0.262, *y* = −4.503 + 2.007 × ln (age) ± 0.218, *y* = −4.860 + 2.117 × ln(age) ± 0.200 for lateral, middle, and medial ends, respectively), linearly with respect to its projection surface area (*y* = −31.390 + 2.432 × age ± 4.599), and fourth-degree polynomially with respect to its volume (*y* = 28.161 + 0.00017 × (age)^4^ ± 15.357).

In the professional literature, there are no reports concerning the dimensions of the humeral shaft ossification center, which precludes a more comprehensive discussion in this subject. The dimensions of the humeral shaft ossification center obtained in the present study may be critical in diagnosing skeletal dysplasias that are often characterized by a disrupted or completely halted growth of the humerus in the fetus. Disproportionate nanism with short limbs can be a result of shortened proximal parts of limbs, i.e., the humerus and femur (rhizomelia). Dysplasia affecting the shafts of the long bones causes their enlargement, sclerotization, thickening of the cortical layer, and thinning or enlargement of the medullary cavity. Abnormalities in the long bones may also be accompanied by abnormalities in the spinal cord, i.e., spondylodysplasia. Achondrogenesis and thanatophoric dysplasia are lethal with a typical image of hypoplasia of the long bones of the upper limbs, including the humerus [7, 8, 13, 34]. Fetal skeletal dysplasia affects approximately 2.4–4.5 out of 10,000 births [2]. Of note, Down’s syndrome is the most frequent chromosomal abnormality observed at birth. According to Benaceraff et al. [4–6], the coexistence of shortened femur or humerus with a thickened nuchal fold is a good predictive sign for the trisomy 21. Stempfle et al. [30] observed the tendency of slowed growth of the limb long bones during the third trimester more pronounced in the trisomic group than in the normal fetuses.

The very first diagnosis of suspected skeletodysplasias is usually stated during routine prenatal ultrasonic examinations. Unfortunately, the sensitivity of ultrasound when diagnosing skeletal dysplasia is only 40–60%. Sonographic findings usually include foreshortened long bones for gestational age, abnormal skeletal morphologic features, abnormal mineralization, and fractures. Therefore, ultrasonography alone is unsatisfactory to diagnose such deformities like narrow thorax, bowed long bones, or more complex abnormalities [22]. Victoria et al. [32] compared both the effectiveness and utility of ultrasonography and CT in the prenatal diagnostics of skeletodysplasias. The authors demonstrated that among the 21 cases included in the study, in only 5 cases CT and ultrasonic findings were equivalent, while in 17 cases CT unveiled novel osseous findings, invisible by ultrasound. Among 218 measurements performed, a total of 4 erroneous findings referred to CT, and as many as 19 erroneous findings referred to ultrasonography. Cassart et al. [10] compared 2D US and 3D CT methods when diagnosing skeletal dysplasias in 11 fetuses aged 26–36 weeks. It is noteworthy that the total CT dose index was 3.12 mGy. As it turned out, the correct diagnosis was done in 2 cases using 2D US and in 8 cases using 3D CT. Thus, the authors found 3D CT to be more accurate than 2D US in the diagnosis of fetal skeletodysplasias.

Of course, the analysis of fetal CT images has some limitations, since there are some areas that require further investigation of the fetal skeleton, such as long bone lengths on CT images at different gestational ages. Besides, when compared to ultrasonography, the CT evaluation of fetal bone mineralization is more difficult as there have been no standards available yet. The visualization of the fetal hands and feet is also limited at earlier gestational ages, and as late as in the late second and third trimesters their images are satisfactory. The advantage of fetal CT examinations results from the fact that it can be entirely reinterpreted at any given time with no loss of imaging details after the study is finished [32]. Furthermore, CT examinations can discriminate one skeletal dysplasia from another in terms of impact and long-term outcome [22]. The American College of Radiology recognized a dose of less than 50 msV as no risk to the pregnant women and *in utero* fetus. McCollough et al. [24] even claimed that at a dose of 100 msV, the absolute risk of fetal effects was small, and at a dose of 50 msV it was just negligible. To our opinion, it should be emphasized that CT examination cannot be used in the evaluation of minor osseous abnormalities. Instead, it may be performed as a complementary method to ultrasonography in the diagnosis of severe and potentially lethal abnormalities. As reported by Macé et al. [22], in the diagnosis of fetal skeletodysplasias a helical CT examination is useful from week 26 of gestation and should be performed in cases with severe micromelia below the 3rd percentile and for those ≤10th percentile associated with another bone sign. According to these authors, the fetal age above 26 weeks is a period of pregnancy which ensures additional safety because of the development of potential exposed organs. In the third trimester of pregnancy, the ossification is satisfactory to correctly analyze CT images. Simultaneously, it is more difficult to obtain adequate viewing planes in tridimensional ultrasonography.

The main limitation of the present study has resulted from a relatively narrow fetal age, varying from 17 to 30 weeks of gestation and the small number of cases, including 48 human fetuses. Another partial limitation may be that all measurements were performed by one observer in a blind fashion.

## Conclusions

1. The morphometric characteristics of the humeral shaft ossification center display no sex differences.
2. The ossification center of the humeral shaft grows logarithmically with respect to its length and transverse diameters, linearly with respect to its projection surface area, and fourth-degree polynomially with respect to its volume.
3. The obtained morphometric data of the humeral shaft ossification center are considered normative for respective prenatal weeks and may be of relevance in both the estimation of fetal ages and the ultrasonic diagnostics of congenital defects.
